# Are Educators Actually Coaches? The Implication of Teaching and Learning via Simulation in Education in Healthcare Professions

**DOI:** 10.7759/cureus.734

**Published:** 2016-08-11

**Authors:** William C. I Janes, Dustin Silvey, Adam Dubrowski

**Affiliations:** 1 Human Kinetics and Recreation, Memorial University of Newfoundland; 2 Emergency Medicine, Pediatrics, Memorial University of Newfoundland; 3 Marine Institute, Memorial University of Newfoundland

**Keywords:** coach, educator, simulation

## Abstract

Simulation is a unique pedagogical tool designed specifically to develop skills, attitudes, behaviors, and knowledge using experiential learning. Though the teachers in the field of simulation are known as educators, they are generally categorized as educators or coaches and must employ unique pedagogic approaches. Though the aspects of educating and coaching are similar, there are numerous differences that set the two roles apart. Thus, the purpose of this editorial is to highlight the differences between the two roles and also to contextualize their differences, as they relate to simulation in healthcare professions, teaching, and learning. The fundamental proposition of this editorial is to highlight that the teachers who use simulation as their teaching and learning technology function as coaches and not educators as they are currently labeled. Like Haji et al. propose in their article titled "What we call what we do affects how we do it: a new nomenclature for simulation research in medical education," we propose that there needs to be a slight shift in the nomenclature of simulation.

## Editorial

### Introduction

Teaching, when done well, is one of the most important and rewarding careers. To teach well, a teacher requires both subject expertise and the skills to convey the knowledge to the learners [1]. It is likely that most of us have experienced various permutations of teaching abilities throughout the years; including being taught by teachers who are knowledgeable and are able to teach, being taught by those who are knowledgeable but are unable to teach, being taught by teachers who are not so knowledgeable but are able to teach, or being taught by teachers who are neither knowledgeable nor able to teach. 

Teaching generally comes from individuals who are either educators or coaches. Although there are a lot of similarities between educating and coaching, there are numerous differences between the two that set them apart. Thus, the purpose of this editorial is to highlight the differences between the two roles, to contextualize their differences as they relate to simulation-augmented teaching and learning in healthcare professions, and to propose a slight shift in the existing simulation nomenclature. The impetus for this editorial comes from an article by Haji, et al. [2] entitled “What we call what we do affects how we do it: a new nomenclature for simulation research in medical education.” Accordingly, here we argue that individuals who use simulation for teaching purposes may adopt the role of an educator rather than the more appropriate role of a coach.

### Simulation

Simulation is the imitation of the operation of a real-world process or system over time [3]. To successfully simulate a process or a system, a model (or simulator) that represents the key characteristics or behaviors one wishes to simulate must be developed [4]. The simulator represents the process or the system, whereas the simulation represents the operation of the system over time and how it relates to the other systems. Consequently, the act of teaching using a simulator, i.e. simulation-augmented pedagogy, requires a process whereby a learner acquires actions, behaviors, and skills by interacting with the simulated system or with a set of systems over time. For example, in a healthcare profession, educating a patient is considered as a system. It can be represented by a computerized mannequin that is capable of behaving in a similar way to a human being. Changing the physiology of this simulator over time in response to the learners' action in an effort to elicit specific knowledge skills or attitudes is the act of simulation.

### Educators

An educator is an individual who plays a multifaceted role and can be characterized as a teacher or an administrator [5]. The main goal of an educator is to educate. It is a process designed to impart intellectual, moral, and social instructions to a learner [6]. Educators are individuals who have received post-secondary training in the principles and theories of education at various stages and who facilitate learning in a given subject area [7]. The main goal of an educator is to relay knowledge to learners and at the same time adhere to the guidelines set by the school administration [1]. Educators are expected to test their learners' knowledge and their retention capacity of a set subject matter [1]. The way in which the educators conduct themselves and teach the curriculum impacts both the learning experience and the motivation, which in turn contributes to the learners' success [1]. Learners spend a significant amount of time gaining new knowledge from their educators. Thus, the way the educator presents the material and structures the learning environment can have an enormous impact [1]. Educators play an important role in the lives of their learners as they impart knowledge under a set curriculum and are the role models shaping their learning environment.

### Coaches

Like educators, coaches also play a multifaceted role—they teach and train athletes and/or performers [8]. Coaches receive limited formal training, often in the form of short coaching clinics or seminars provided by experienced coaches [9]. However, their vast experience can replace, and in some cases, be superior to the formal training that is provided to educators. Furthermore, coaches are given more freedom in determining how to structure their role as a teacher [9]. For example, some coaches may emphasize skill development, while others may emphasize the need for team cohesiveness [9]. The role of a coach includes many responsibilities like providing practice plans and implementation, motivating learners for higher performance, and creating cohesiveness within a team environment [9]. Coaches are teachers within their respective areas of expertise and play an important role in training individuals for a given sport or task. The concept of coaching is based on assisting individuals in achieving their own goals and maximizing their potential [10]. Coaching generally involves overseeing a task and occasionally providing feedback on how to perform the given task more efficiently. However, coaching is not instructing the individuals on what to do and how to do it [10]. Training is provided through instructions, performance analysis, encouragement, and feedback that helps the individuals to become more effective in the act they are attempting to master [7, 9]. Coaches must always perceive new ways to present their new knowledge to each individual in order to provide the greatest learning experience. A coach must create the right conditions to influence the learning and to motivate the individuals to achieve their goals, irrespective of whether they are independent or are a member of a team [9].

### Comparison

Educators and coaches share a number of similarities within their positions. Educators are role models for learners at all levels, providing guidance and teaching life lessons from their experiences. Coaches also act as role models and mentors for their athletes through their willingness to invest time in their learners [11]. For both the roles, teaching is the ultimate goal and both relay knowledge and expertise to their respective learners.

However, there are also many differences between the two roles, including the depth of knowledge, knowledge transfer, and feedback mechanisms. Educators place an emphasis on cognition and the knowledge that educators impart includes a wide array of theories [7]. The knowledge delivered by coaches emphasizes other domains such as psychomotor and psychosocial skills and is related to the mastering of a skill by doing. Educators often communicate to learners through lecture-based means [1] and provide feedback through tests of material/concept comprehension. On the other hand, coaches provide individualized feedback that is often derived from intimate familiarity with the knowledge, skills or attitudes embedded in their teaching.

Finally, what sets the educators and coaches apart in their teaching styles are the learners' involvement. An educator is seen as a central figure in a learner's education, having total control over the learner in the classroom irrespective of the learner's wish, [1] whereas a coach welcomes inputs from the learner about his/her decision on learning. They promote learning rather than lecturing [1].

### Conclusion

The impetus for this editorial was an article published by Haji, et al. [2] entitled “What we call what we do affects how we do it: a new nomenclature for simulation research in medical education.” Here we argue that calling individuals who use simulation for teaching purposes *educators* may result in the adoption of an *educator* role rather than that of a *coach*.

Recognizing that there are a number of similarities in these roles and keeping in mind the differences, the question we pose to individuals who teach using simulation is whether they consider themselves educators or coaches.
